# The field monitoring experiment of the high-level key stratum movement in coal mining based on collaborative DOFS and MPBX

**DOI:** 10.1038/s41598-021-04578-w

**Published:** 2022-01-13

**Authors:** Xiaozhen Wang, Jianlin Xie, Weibing Zhu, Jialin Xu

**Affiliations:** 1 State Key Laboratory of Coal Resources and Safe Mining, China University of Mining and Technology, Xuzhou, China; 2 School of Mines, China University of Mining and Technology, Xuzhou, China

**Keywords:** Energy science and technology, Engineering

## Abstract

The deformation and movement characteristics of high-level key stratums in overlying strata are important for estimating ground subsidence and understanding failure characteristics of ultrathick strata during mining. In this study, a distributed optical fiber sensor (DOFS) and multipoint borehole extensometers (MPBXs) were collaboratively employed to monitor the deformation of high-level key stratums in situ during the mining process at working face 130,604 of the Maiduoshan Coal Mine. DOFS monitoring results showed that the distance from advance influence of mining on the ground surface is 219.2 m. The deformation of the shallow stratums were greater and was affected earlier than that of the deep stratums. The deformation in the strata did not occur continuously and the boundary curve of the impact from advance mining was not a straight line with the advancement of the working face. By the MPBX technology, we measured the strata movement and obtained four-stage characteristics of high-level key stratum movement. The subsidence of the primary key stratum and the sub key stratum were monitored to reach 1389 and 1437 mm; their final relative displacement differed by 48 mm. No bed separation was observed in between the strata, and the key stratums tended to sink as a whole with the advancement of the working face. This research guides the analysis the movement of thick high-level key stratums.

## Introduction

Western China has ideal geological conditions to support coal mining. Abundant reserves of extra-thick coal seams with a thickness of 6–20 m or more have been discovered in that region. According to relevant statistics, the total coal production capacity from Shanxi, Shaanxi, and Inner Mongolia Provinces has reached 2.183 billion tons in 2019, which accounts for 62.5% of the total coal production capacity in China. However, many newly constructed or existing coal mines in operation in western China have frequently encountered dynamic disasters during the mining of extra-thick coal seams in the past 15 years. These disasters, including support crushing in the working face^1,2^, rock burst^3^, roof bed separation water disaster^4^, have seriously affected the safety and efficiency of the operation of the coal mines.

For example, stratums with a thickness of 50–100 m were found to exist at 150–180 m above the roof of the coal seam in the Binchang mining area (Tingnan Coal Mine, Shaanxi Province), which have caused more than 40 rock burst accidents in roadway during the mining process at the No. 206 working face in the second panel^5,6^. Similarly, ultrathick sandstones with a thickness of 40–100 m were found to exist in the overlying strata at 200 m above the coal seam in the Maiduoshan Coal Mine, which results in vibrating ground and serious deformation of the underground roadway along the gob during the mining process at the working face. The traditional theory of mining pressure focuses on the analysis of strong mining pressure and support crushing disaster caused by the fracture of the key stratums at a short distance from the coal seam^7–9^. However, the problem of abnormal mining pressure caused by the ultrathick key stratum far above the coal seam has not been systematically studied. In fact, the movement of the ultra-thick rock formation at a distance that is 10–20 times the mining height above the coal seam will still have a significant effect on the mining pressure. Zhu^10^ proposed a structural mechanics model of far-field key stratums in overlying strata at a large coal mine. Such model provides a preliminary explanation for the long-term occurrence of high mining pressure. Li et al.^11^ performed a theoretical analysis to investigate the breaking length of ultra-thick rock formations, the characteristics of strain energy conversion, and their effect on roadway deformation. Xuan et al.^12^ investigated coal and gas outburst during the mining process at a long-distance roadway in the presence of an ultrathick (120 m) igneous rock formation at 180 m above the coal seam. Han et al.^13^ analyzed the distribution of the mining-induced stress under an ultrathick stratum and found that the distribution of the mining-induced stress will be affected by the special structure of the overlying strata. Above studies revealed the abnormal distribution of support stress under ultrathick rock formation, wherein no deformation occurred and movement monitoring was not required.

Several other studies attempted to reveal the relationship between high-level stratum movement and underground pressure by combining different approaches, including surface drilling and monitoring of the underground mining pressure^14–16^. However, these studies did not succeed in providing detailed information about the movement inside the stratum. He et al.^17^ analyzed the shape of the boundary curve of rock movement based on theoretical analysis and simulations, which provided a reference to predicting the subsidence range; however, they have not been validated experimentally yet. In order to analyze strata movement and deformation, micro seismic technology was used to monitor rock strata for rupture signals^18–20^. While it is difficult to quantitatively determine the extent of the motion-induced damage using this method, and the scope of monitoring is limited, especially the high-level strata in deep mining.

Distributed fiber optic sensing can be used to increase monitoring depth, this technique is best suited to areas wherein strata movement is relatively small. Cappa et al.^21^ used fiber optic cables to characterize highly heterogeneous elastic displacement fields in fractured rocks. However, fiber optic cables can snap when the working face passes through the monitoring boreholes, or when the strata movement and deformations are large, leading to data loss. By installing three types of fiber optic lines in two boreholes, Liu et al.^22^ studied the deformation and damage of mining-perturbed strata and found that fiber optic lines at different depths were deformed in different ways. The multipoint borehole extensometer (MPBX) is an instrument that can be used for large displacement monitoring. Ingram et al.^23^ studied the overburden movement that was caused by the mining of a longwall panel—with a 270 m-wide face, buried depths of 95–105 m and an extraction thickness of 1.8 m—by setting up monitoring lines with MPBX in the overburden. The MPBXs were installed by injecting grout into the boreholes. However, it is significantly more difficult to drill boreholes or monitor strata movement at large buried depths. Holla et al.^24^ planned to use a multi-wire borehole instrumentation system to monitor strata movement and changes in strata permeability during the repeated mining of multiple working faces, at a monitoring depth of 424 m. However, due to the difficulty of drilling such a deep borehole, they were only able to install their instruments at a depth of 165 m. Despite the efforts of researchers, most of the strata movement monitoring attempts performed thus far have been limited to depths of less than 200 m.

The aforementioned studies have facilitated the analysis of the movement of high-level key stratums. However, very few studies attempted to measure the movement of high-level key stratums in situ because of the limitation of engineering conditions. As a consequence, the obtained results from previous studies lack sufficient validation and are not corroborated with real-world engineering data. To overcome this, herein, the mining-induced deformation and movement of high-level and thick key stratum were monitored on the basis of local mining and occurrence conditions, which provides basic data support for analyzing the failure characteristics of high-level key stratums.

## Basic conditions of working face

### Mining conditions

**Figure 1:**
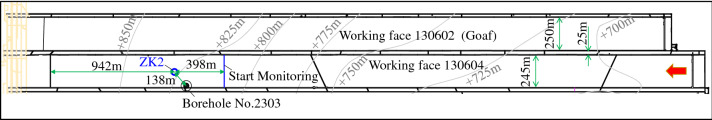
Plan of the working face. Upper working face 130,602 has been mined. The lower working face 130,604 has been mined partly before monitoring.

**Figure 2:**
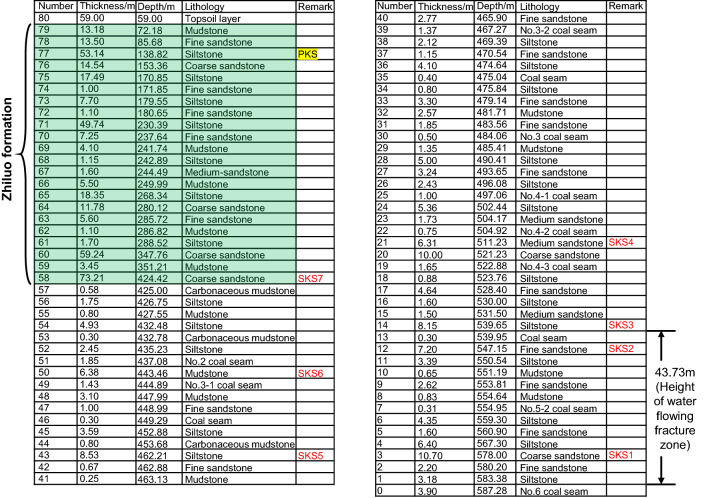
Borehole columnar section and locations of PKS and SKS. Using the key stratum identification method, there are 8 key stratums in overburden. Only the shallowest one is PKS, the others are all SKSs. SKS can control the movement of the upper strata up to the adjacent upper SKS. For example, SKS1 can control stratums numbered 4–11, and SKS3 can control stratums numbered 15–20. PKS is the shallowest key stratum in overburden. PKS can control stratums all above it, it is numbered as 78–80.

The Maiduoshan Coal Mine is located at the southernmost end of the Yuanyang Lake Mining Area. The bedrock of the Maiduoshan mine field consists of Jurassic strata. Working face 130,604 in the Maiduoshan Coal Mine, which is the second working face in the No. 13 mining area, is in charge of coal extraction from No. 6 coal seam. This working face has a width of 245 m, a strike length of 4962 m, an average mining height of 3.8 m, an average dip angle of 2°, an average burial depth of 612 m, and a surface soil thickness of 60 m. The hydraulic-powered supports that are used in this working face has a rated working resistance of 10,000 kN. The neighboring working face (i.e., working face 130,602) is the first working face in the mining area that has a width of 250 m and a strike length of 4717.9 m. These two working faces are separated by coal pillar of 25 m width. Figure 1 shows the plan of the working face. The Zhiluo formation is the main aquifer in the local coal mine. This formation is widely distributed in the entire coal mine with an average thickness of 231.11 m. The distance between the bottom interface of Zhiluo formation and the coal seam is approximately 160 m. The formation consists of grey and greyish green sandstones with fine, medium, and coarse grain sizes. Fractures/fissures have also been found in certain local regions of the formation. In general, the aquifer in Zhiluo formation exhibits good permeability and a strong water yield property. After considering the geological data obtained from borehole No. 2303 in working face 130,604 as an example (Fig. 2), the thicknesses of the Zhiluo formation, surface soil layer, and coal seam are 365.42, 59, and 3.9 m, respectively.

### Key stratums in overburden

According to ‘‘Key Strata Theory’’ introduced in the literature^25^, the stratum that controls the movement of the whole or partial overburden strata is defined as the key stratum (KS), that is, when the KS breaks, the whole or a part of the overburden strata above the KS will subside simultaneously, the former is defined as the primary key stratum (PKS), and the latter the sub key stratum (SKS). There may be more than one KS in the overburden strata; however, there is only one PKS. The strata behavior is directly controlled by the break and movement of KS. This theory has been widely used in China in the field of strata behavior control. The method of critical stratum discrimination is mainly used to determine the critical stratum. The bearing capacity and rupture distance of different rock strata were calculated based on the thickness and strength of rock strata. The location of hard rock strata with collaborative deformation was determined according to the bearing capacity, and the rupture distance of hard rock strata was determined according to the rupture distance of the hard rock strata. By using the key stratum identification method, according to the physical information such as stratification, thickness, bulk density, elastic modulus and tensile strength of rock strata in geological histogram, seven key stratums were found in the overlying strata. The main key stratum is a 53.14-m-thick siltstone layer, which is located at 444.56 m from the roof of the coal seam. Multiple layers of thick hard sandstones (> 50 m thickness), i.e., a 59.24- and 73.21-m-thick layers of coarse-grained sandstone were found to exist at the bottom of the Zhiluo formation. Among them, the 73.21-m-thick layer of coarse-grained sandstone located at 158.96 m from the roof of the coal seam was identified to be the key stratum.

Based on the locations of the key stratums, we further analyzed the degree of development for the water flowing fractured zone^26^. Specifically, the height of the water flowing fractured zone in working face 130,604 was calculated to be approximately 43.73 m. The key stratums identified in the Zhiluo formation are located within the continuous bending zone. The distance between the key stratum and the coal seam is more than 40 times the thickness of the coal seam. Therefore, these key stratums are considered to be at a high position in the overlying strata and are called high-level key stratums. The thicknesses of the other strata underneath the Zhiluo formation are generally < 10 m.

### Ground subsidence

During the mining process at working face 130,602 in the Maiduoshan Coal Mine, only a minor level of ground subsidence was observed. One month after stopping the mining process, the maximum subsidence of the surface was measured to be 136 mm. These data suggest that the subsidence has only proceeded slightly during mining. Since the ground subsidence was dominated by the primary key stratum in the overlying strata, the low level of subsidence indicates that thick and high-level key stratum in the overlying Zhiluo formation is only moving at an early stage and may still be under the dangling condition. With working face 130,602 under excavation, the mining operation at working face 130,604 will further increase the total scale of the mining area. In this case, it becomes more difficult to determine if the thick and high-level key stratums in the Zhiluo formation will remain under the dangling condition, and whether large-scale fractures will occur during the mining process at working face 130,604. In other words, it becomes challenging to evaluate the safety risk (e.g., ground collapse or underground dynamic disaster) during the mining process. Therefore, it is imperative to monitor the deformation and moving dynamics of the high-level key stratums in the Zhiluo formation.

## Monitoring methods and instruments

### Monitoring methods

For monitoring the deformation and movement of high-level key stratums, vertical boreholes were drilled from the ground into the Zhiluo formation. Distributed optical fiber sensor (DOFS) monitoring technology and multipoint borehole extensometer (MPBX) methods were combined to obtain the deformation and movement data of the strata.

DOFS has been subjected to rapid development in the past decades. This well-developed rock deformation monitoring technique has already been employed in various different applications, including infrastructural health monitoring, deformation and damage level assessment of overlying rocks, borehole pressure relief evaluation, tunnel deformation measurement, slope stability assessment, and ground subsidence monitoring^27,28^. As the mining operation proceeds forward in the working face, the overlying strata will start to deform, which causes the optical cable sensor (containing the optical fiber) to stretch along the axial direction in the borehole. This stretching behavior allows us to sense the stress distribution inside the strata. By analyzing the temporal and spatial evolution of the strain along the fiber sensor, we can obtain the deformation condition of the overlying rock during the mining process. In this study, DOFS with optical fibers wrapped inside were used for optical monitoring. These optical cable sensors have a diameter of 5.3 mm, a maximum loss of 0.3 dB km, and an allowable tension stress of 2500 N.

MPBX is an instrument used to measure the differential vertical movements of the selected rock horizons in a borehole relative to the surface. Each MPBX unit comprises several measuring points, pressure-resistant hollow plastic cables, steel cables and encoders with tension. Different measuring points were anchored on pre-determined strata inside the borehole. Each steel cable is slipped through the plastic hollow cable and then extended from the measuring point to the surface where it is attached to the encoder. The pressure-resistant hollow cables of MPBX contain steel cable inside, which can withstand an external liquid pressure of 17 MPa, and is suitable for the installation and sealing of large depth drilling hole; it protects the steel cable from contacting and being glued to the grout/bentonite mixture that is used to backfill after the installation of measuring points has been completed. These measuring points will experience the same movement as the strata that they are anchored on. Therefore, the movement data of the pre-determined stratum can be transferred to the encoders located at ground surface through the steel cable. The resolution of MPBX is 0.01 mm and the monitoring frequency is 1 min. Thus, we can obtain the relative movement of stratum. This monitoring information can be transmitted and stored remotely through mobile networks. Subsequently, the absolute movement information inside the strata can be obtained by combining the displacement measurement with the ground subsidence observed at the borehole.

The aforementioned two methods are based on different monitoring principles; however, each has its own advantages. The maximum single point shape variable of DOFS is 20,000 micro-strain. The ultimate elongation of 1 m length optical fiber is 2 cm, which is usually difficult to adapt to large mining deformation. MPBX monitors the subsidence of the anchored rock strata by setting monitoring anchor points in the borehole and connecting them with the surface displacement sensor through the cable. Due to the internal movable steel cable, MPBX can adapt to the movement monitoring of the large movement of rock strata. In this paper, the collaborative method of monitoring deformation before the working face reaches the borehole and the movement that working face surpass the borehole is creatively adopted, which has not been used in previous studies. Therefore, this collaborative monitoring method is novel. Moreover, in terms of the whole process of rock movement caused by coal mining, limited attention has been paid to the advance influence of mining in overburden, and the monitoring activities are even less. This study adopts the monitoring means combined with two methods to monitor the whole process of mining influence from the beginning to the end.

**Figure 3:**
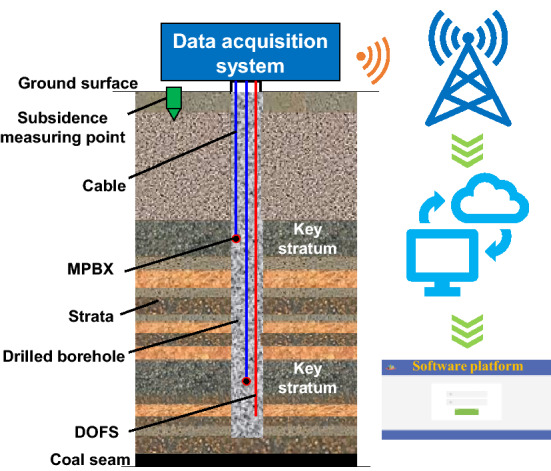
Schematic of the monitoring system.

The monitoring cables were installed and sealed inside the borehole. The strata movement during the mining process was subsequently measured by the monitoring device installed at the entrance of the borehole. Figure 3 shows the schematic diagram of the entire monitoring system.

### Monitoring design

Based on the mining conditions of the Maiduoshan Coal Mine, the monitoring boreholes were arranged in the advance solid coal region, i.e., an area that remains unaffected by the mining operation at working face 130,604. Finally, the borehole was designed at the center of the inclination, 3939 m away from the setup entry, 942 m away from the stop line, 128 m away from the conveyor gateway, and 138 m away from a nearby borehole (No. 2303). The borehole depth is approximately 440 m, and the bottom of the hole is located approximately 15 m beneath the bottom boundary of the Zhiluo formation.

DOFS was installed to monitor the deformation characteristics of all strata inside the borehole during the mining process. The bottom boundary of the ultra-thick Zhiluo formation in the overlying strata is 424 m away from the entrance of the borehole. The maximum monitoring depth of the optical fiber is 437 m.

To obtain the movement data of the high-level key stratums, the MPBX method was used and two measuring points (labeled as measuring points 1# and 2#) were arranged in the high-level key stratums at a depths of 410 and 110 m, respectively, beneath the borehole entrance.

**Figure 4:**
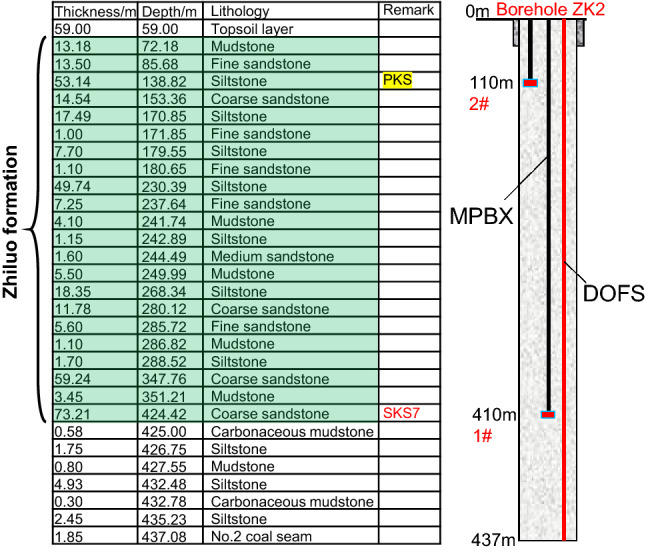
Monitoring plan for strata movement in the Zhiluo formation.

The locations of the monitoring points in the Zhiluo formation in the overlying strata are shown in Fig. 4.

### Monitoring equipment

**Figure 5:**
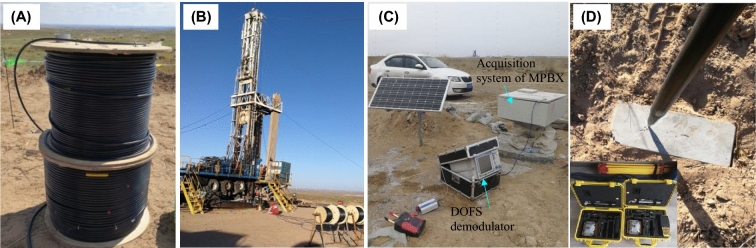
Installation and operation of movement monitoring device inside the borehole. (**A**) Pressure-resistant hollow cables. (**B**) Monitoring device installation. (**C**) In-suit monitoring. Data acquisition system of MPBX on the right. The resolution of MPBX is 0.01 mm and the monitoring frequency is 1 min. DOFS data are collected by the demodulator on the left. (**D**) Subsidence measurement using RTK technology. Subsidence monitoring point is near the borehole. The absolute movement information inside the strata can be obtained by combining the MPBX date observed at the borehole with the ground subsidence.

MPBX was used to monitor the strata movement. Pressure-resistant hollow cables of MPBX contain, steel cable inside it, can withstand 17 MPa external liquid pressure and suitable for the installation and sealing of large depth drilling hole, as shown in Fig. 5A. The monitoring device was placed inside the stratum by using the borehole drilling rig shown in Fig. 5B. After the monitoring device was placed and sealed inside the borehole, the strata movement and optical fiber deformation data were collected by the data acquisition system located at the entrance of the borehole, as shown in Fig. 5C. Finally, the ground subsidence at the entrance of the borehole was recorded while simultaneously monitoring the strata movement inside the borehole, as shown in Fig. 5D. Ground subsidence monitoring is used by real-time kinematic technology (RTK). The monitoring information contain the X, Y and Z movements; however, the vertical movement (Z movement) data is widely used. The collaborative method of monitoring mining-induced advance deformation (before borehole) and strata movement after mining (passed borehole) in the same vertical borehole is creatively adopted in this paper, which has not been reported in any previous studies. Therefore, the collaborative monitoring method itself is innovative.

## Results

### Deformation process of the strata by DOFS

After the monitoring device was sealed on September 29th, 2018, the deformation process of the strata was monitored in real time inside the borehole. The first data were collected by the optical fiber inside the borehole on October 2nd, 2018, and used as the initial value. At this time, the working face was located 368 m away from the borehole. Afterwards, the movement data were collected periodically as the working face advanced continuously. The monitoring interval was adjusted according to the distance between the working face and the borehole; such time interval can be as long as 4–7 d, and as short as 0.5–1 d. The strain variation can then be obtained by subtracting the initial value from the monitoring result.

**Figure 6:**
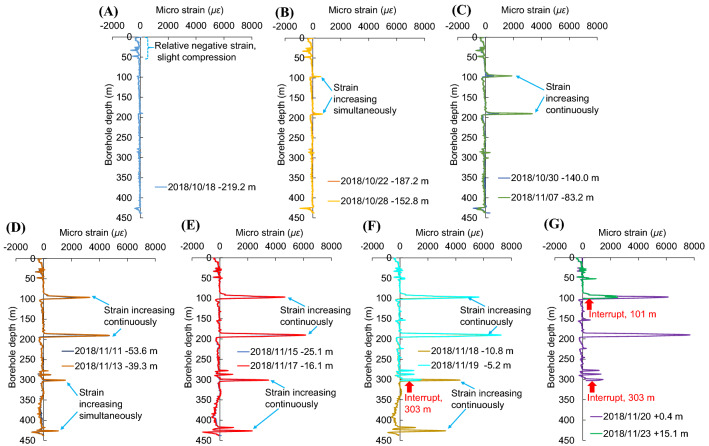
Change of the relative strain of the optical fiber in the borehole. The horizontal axis is the relative micro-strain of the optical fiber. A positive value indicates that the measured strain is larger than the initial value, such that the strata is under tension. Conversely, a negative value indicates that the measured strain is smaller than the initial value, such that the strata is under compression. The vertical axis is the depth of the borehole. A negative distance represents the distance of borehole ahead of the working face in the mining advance direction, while a positive distance represents the distance of the working face ahead of the borehole. (**A**) Occurrence of compression in surface soil. (**B**) Initiation of deformation in shallow strata. (**C**) Increasing deformation in shallow strata. (**D**) Initiation of deformation in deep strata and increasing deformation in shallow strata. (**E**) Increasing deformation in both shallow and deep strata. (**F**) Interrupt till a depth of 303 m. (**G**) Interrupt till a depth of 101 m.

Figure 6 shows the change of the relative strain of the optical fiber as the working face approaches the borehole. As the working face approaches the borehole, the positive strain induced by the mining process is found to be much stronger in the shallow stratum than that in the deep stratum. Additionally, the strain was found to concentrate at a few locations, such as a depth of 87–103, 185–196, 275.8–305, and 416.4–430 m in the borehole. The peak strain value was measured at a depth of 97.1, 190.5, 301.4, and 426.5 m in the borehole. These results suggest that advance mining has a stronger impact on the shallow strata than on the deep strata.

On October 18th, 2018, the borehole was 219.2 m ahead of the working face. The surface soil (59 m beneath the entrance of the borehole) is affected by compression deformation while no significant change was observed in other deeper regions (Fig. 6A), which can be considered that the distance from advance influence of mining on the ground surface is 219.2 m. On October 25th, the borehole was 168.8 m ahead of the working face, and a slight increase in strain was observed in the shallow region. On October 28th, the borehole was 152.8 m ahead of the working face. The relative strain of the optical fiber started to increase significantly accompanied by the initiation of deformation at a depth of 97 and 195 m (Fig. 6B).

On October 30th, the borehole was 140.0 m ahead of the working face. The relative strain of the optical fiber continued to increase at the two locations mentioned above, but no significant strain change in the deeper region (Fig. 6C). On November 7th, the borehole was 83.2 m ahead of the working face. The strain at the two aforementioned locations continued to increase at a faster rate while the deep strata is still deformation-free. In other words, the deformation did not proceed downwards in the borehole during the forward advance of the working face over 69.6 m (from 152.8 to 83.2 m ahead of the working face) when the deformation of the two aforementioned locations started (Fig. 6C). Instead, the deformation strain kept accumulating at the original location where it occurred. This feature suggests that the influence of advance mining increases nonlinearly in the overlying strata.

On November 11th, 2018, the borehole was 53.6 m ahead of the working face. The relative strain started increasing in the strata at a depth of 300 and 426 m simultaneously. These two locations were 126 m away from each other in the vertical direction. At the same time, the relative strain at a depth of 97 and 195 m continued to increase (Fig. 6D). As the working face was approaching the borehole, the stress kept increasing at all four locations in the overlying strata. This feature is demonstrated by the data obtained on November 17th when the borehole was 53.6 m ahead of the working face (Fig. 6E). On November 19th, the borehole was 5.2 m ahead of the working face. The strain kept increasing in the shallow region, but a sudden interruption was observed in the lower section of the optical fiber. The lowest point that can still be monitored by the optical fiber rose from a depth of 437–303 m. No signal was received below the interruption position (Fig. 6F). On November 20th, the working face was 0.4 m ahead of the borehole and the lowest measurable point was still at a depth of 303 m. On November 23rd, the working face was 15.1 m ahead of the borehole and the position of interruption in the optical fiber rose to a depth of 101 m (Fig. 6G). No further significant change in the interruption position was observed afterwards.

### Relationship between relative deformation and strata distribution

**Figure 7:**
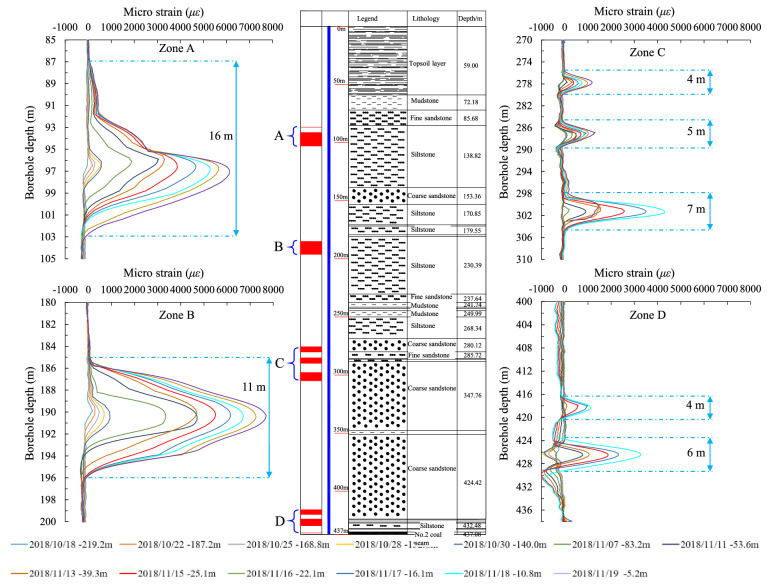
Relative strain of the optical fiber and corresponding stratum column.

In order to explain the stepwise change of relative strain, the four regions of significant strain change were magnified from the shallow to the deep area, and further labeled against the borehole column (Fig. 7). As shown by our analysis, the tensile deformation induced by advance mining was mostly found in the upper region of thick stratum in the formation (Zone A and Zone B), and the interface between the thick and thin (or weak) stratums (Zone C and Zone D). These locations include (Zone A) the upper region of the primary key stratum in the overlying stratum (at a depth of 91.7–102 m), (Zone B) the upper region of the 49.74-m-thick siltstone layer in the overlying stratum (at a depth of 185–196 m), (Zone C) the interface between the upper region of the 59.24-m-thick coarse sandstone layer(at a depth of 298.1–305 m) and thin weak stratum (at a depth of 276–290 m)in the overlying stratum, and (Zone D) the interface between the bottom face of the 73.21-m-thick key coarse sandstone layer and the top face of the thin weak stratum at a lower position (at a depth of 424–430 m). The maximum deformation was observed at a depth of 426.5 m, which is close to the bottom boundary of the Zhiluo formation.

As the strata are generally under compression during the advance mining process, a tensile strain indicates that a shear strain exists between different strata, particularly at the interface between the thick and hard strata. Therefore, if the shear strain caused by advance mining is significantly large in between different strata, the horizontal displacement and fracture should also be taken into consideration, such as the shear failure of the vertical borehole.

### Displacement of key stratum with relative to the ground by MPBX

**Figure 8:**
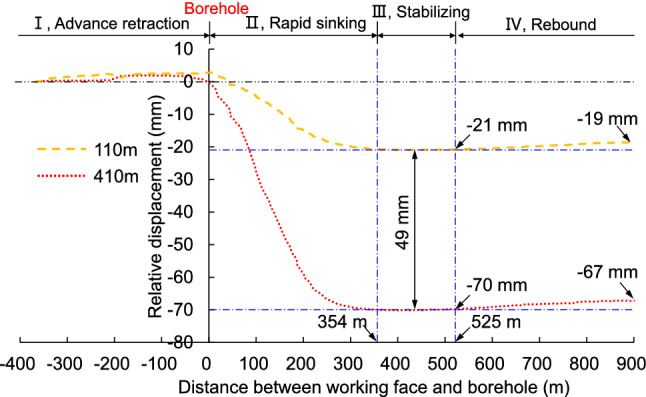
Monitoring results of stratum displacement in the borehole with respect to the ground. The horizontal axis is the distance between the working face and the borehole. Point 0 marks the location of the monitoring borehole, a negative value represents the distance of the borehole ahead of the working face, and a positive distance represents the distance that the working face surpassed the borehole. Because the monitoring device is located at the entrance of the borehole, the vertical axis represents the relative displacement of the measuring point in the borehole along the vertical direction with respect to the entrance of the borehole. The change in relative displacement is 0 at the beginning (when the MPBX was first installed)A negative value indicates that the key stratum is sinking with respect to the entrance of the borehole while a positive value indicates that the opening of the borehole was subsiding due to strata compression.

Figure 8 describes the relative displacements of the MPBX in the borehole with the advancement of working face. This movement may be divided into four distinct stages, which are described as follows.

#### Advance retracting stage

During this stage, the strata will experience advance retraction under the influence of the mining process. Before the working face surpasses the borehole, a slight level of continuous retraction was observed and the total retraction amplitude was approximately 2–3 mm. This retraction shows that the strata between the measuring points and the entrance of the borehole will be affected by the advance mining operation. Specifically, the change in the abutment pressure will cause the strata at measuring points 410 m and 110 m to experience different levels of compression, which further results in a retracting behavior. Finally, the advance retraction phenomenon that is observed using MPBX is consistent with the general compression behavior of the strata recorded by the optical fibers under the influence of advance mining.

#### Rapid sinking stage

The measuring point at a depth of 410 m started to sink when the borehole is 53.6 m ahead of the working face, and rapid sinking stage began when the working face reached the borehole. The monitoring point at a depth of 110 m started to sink when the working face surpassed the borehole by 5.3 m. This sinking phenomenon will cause relative displacement between different strata. As a consequence, the optical fiber in the borehole will experience interruption due to its incapability to withstand tensile deformation. When the working face surpasses the borehole, a relative displacement occurred continuously between the stratum on which the measuring point was located and the ground surface.

#### Stabilizing stage

The relative displacement between the measuring point located at a depth of 110 m on the primary key stratum and the entrance of the borehole reached a maximum value of 21 mm when the working face surpasses the borehole by 367.3 m. Afterwards, the magnitude of the relative displacement remains constant, which marks the start of the stabilizing stage. Similarly, the relative displacement between the measuring point located at a depth of 410 m on another key stratum and the entrance of the borehole will reach a maximum value of 70 mm when the working face surpasses the borehole by 395.6 m. This moment indicates the start of the stabilizing stage for the second measuring point. As shown by these data, the movement of the strata located at a depth between 110 and 410 m (total strata thickness is 300 m) becomes stable when the relative displacement reached approximately 49 mm.

#### Rebound stage

The monitoring data started to rebound with a small amplitude when the working face surpassed the borehole by 897 m. Specifically, the relative displacement of the measuring points located at a depth of 110 and 410 m were reduced by 2 and 3 mm, respectively, compared to the values measured at the stabilizing stage. The magnitude of rebound is close to the magnitude of retraction that occurred during stage I. This feature suggests that the advance abutment pressure no longer affects the stratum monitored in this study after its displacement becomes stable. As a result, a rebound of compression occurs in the stratum, and the final relative displacement is 48 mm.

### Absolute movement of high-level key stratums by MPBX

**Figure 9:**
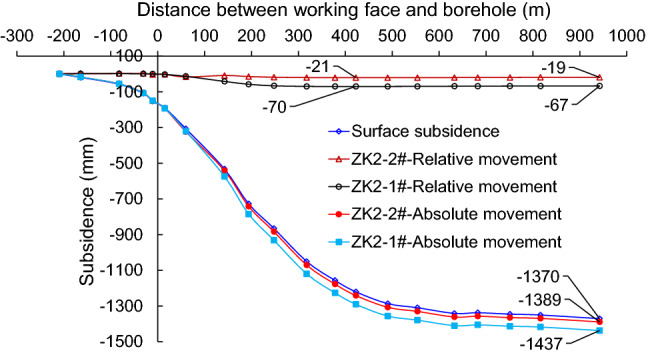
Monitoring results of stratum movement inside the borehole. The horizontal axis is the distance between the working face and the borehole. Point 0 marks the location of the monitoring borehole, a negative value represents the distance of the borehole ahead of the working face, and a positive distance represents the distance that the working face surpassed the borehole. The vertical axis represents the absolute subsidence of high-level key stratums. Absolute subsidence is equal to relative subsidence plus surface subsidence.

The absolute movement of the stratum can be obtained by combining the relative movement data with the ground subsidence measured at the entrance of the borehole, as shown in Fig. 9. When the working face surpassed the monitoring borehole, the stratum started to sink with respect to the entrance of the borehole, as shown by the monitoring data collected at multiple points inside the borehole. Under the influence of the mining operation, the entrance of the borehole underwent a subsidence of approximately 1370 mm. The relative movement inside the stratum with respect to the hole entrance was found to be relatively small compared to the magnitude of ground subsidence. The absolute movement can be obtained by summing the relative movement and the ground subsidence. Specifically, the absolute movement of the two key stratums at a depth of 110 and 410 m were calculated to be 1389 and 1437 mm, respectively, during the advance of the working face.

## Discussion

### Stage-wise deformation of the strata

**Figure 10:**
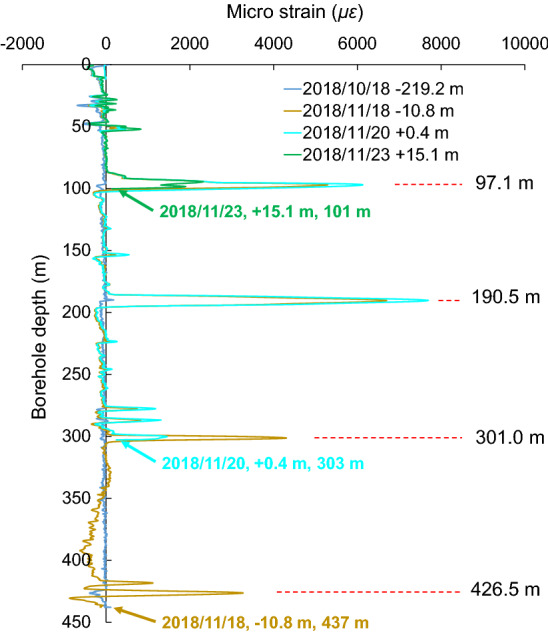
Relative strain of the optical fiber inside the borehole. The data near the arrow represents date, distance of working face ahead of the borehole and the interrupted depth.

Based on the change in the relative strain, we found that the advance mining operation has a stage-wise and non-continuous impact on the strata. This appeared gradually from the shallow to the deep strata. The relative strain of the optical fiber inside the borehole is shown in Fig. 10. The stratums were generally under compression before the working face reached the borehole. After the working face surpassed the borehole, the optical fiber was interrupted by a sudden tension or shear force when the working face approached the borehole and extended towards the shallow region rapidly. The experimental measurements demonstrated that the optical fiber can be used to monitor small deformations of the stratum during advance mining. However, owning to its poor capability to withstand vertical tensile deformation, the optical fiber is not suitable for measuring large deformations of the stratum after the working face surpasses the borehole.

As shown by the monitoring results, the change in strain, which is induced by advance mining, occurred earlier and more strongly in the shallow strata than that in the deep strata. In addition, the impact of advance mining on the strata did not extend linearly from the shallow toward the deep strata during the advance of the working face. Instead, the deformation accumulated at specific layers in the strata. The overlying strata in front of the working face are not affected by the boundary curve of strata movement (or the advance moving line) in a straight-line fashion. In fact, significant obstructions were observed at different locations, which manifests a piecewise characteristic of strata movement boundary.

### Boundary shape of strata movement

**Figure 11:**
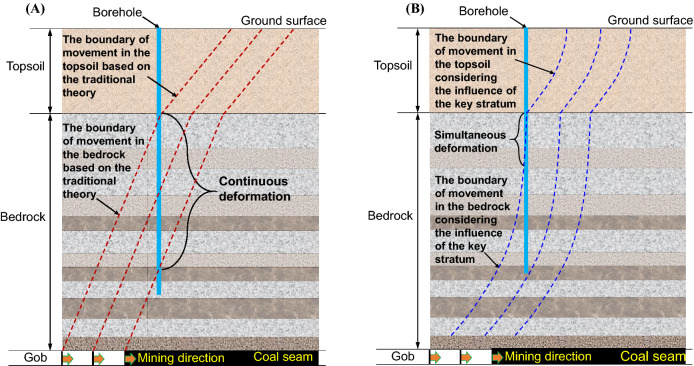
Shape of boundary curve of strata movement during the mining process. The three dash lines represent the boundary of movement in the overburden as the working face advances different locations. Boundary (**A**) of movement in the overburden (**A**) based on the traditional theory and (**B**) considering the influence of the key stratum.

According to the traditional theory of ground subsidence, the boundary of advance strata movement is a straight line that moves at a certain angle during the mining process. In addition, the inclination of the boundary line in the topsoil is significantly different from that in the bedrock (Fig. 11A). The two different boundary curves are joined at the interface between the bedrock and topsoil. As shown by the boundary curve of strata movement in the bedrock and topsoil considering the influence of key stratums (Fig. 11B), the boundary curve of strata movement gradually converges to the vertical direction after mining with the decrease of buried depth of rock strata, that is, the shallow stratums in the bedrock are affected by the mining process while the deep stratums are unaffected by coal mining.

As shown by the field measurement results of DOFS, the mining operation at the working face will have a significant impact on the advance deformation of the overlying strata. In particular, the deformation of the overlying strata caused by advance mining was found to occur first in shallow strata. However, this deformation did not extend linearly towards the deep strata during mining advance. In fact, when the working face reaches a certain distance away from the borehole, the sudden increase in the strain was observed at a new location in the deep strata. This finding suggests that the angle of impact from advance mining is not a constant, and the boundary curve of the impact from advance mining is not a straight line with a fixed inclination. Therefore, under the influence of advance mining, there is a period of time during which the shallow strata experience continuous deformation while the deep strata are free from significant deformation in the bedrock. The theoretical analyses in existing literature are validated to a certain extent by the monitoring results obtained from the engineering tests.

Owning to the limited number of boreholes drilled in this study, we were only able to analyze the deformation of the strata directly above the working face during the mining process. The deformation of the strata above the solid coal pillar outside the gob and the working face, and how the mining process affects this deformation were not explored in this study. These questions will be further investigated in our future work.

### Conditions of movement and fracture inside the strata

The occurrence of displacement difference between different regions in the strata is often considered to be the manifestation of separating fissures between different rock layers. However, it is sometimes difficult to observe the significant separation of fissures by borehole monitoring and in situ detection. As shown in a previous study^29^, the strata are subjective to an accumulative effect of expansion induced by stress relief during the mining process. The maximum expansion of the sandstone layer can reach 1.35–2.19% (average percentage = 1.77%) of its thickness. According to the monitoring results obtained in this study, there is a displacement difference between the measuring points at a depth of 110 and 410 m, the 300 m thick strata expanded by 48 mm due to stress relief. This expansion magnitude is much smaller than the maximum expansion limit (300 m*1.77%, 510 mm), so the internal space of the stratum is still under compression and free from any bed separation. No sudden change was observed in the monitoring data, which suggests that the movement of the thick and high-level hard key stratums is a continuous and slow process during the advance of the working face.

According to existing literature^30^, if sufficient mining progress has been made in the strike direction, fractures may occur in the thick and high-level hard key stratums when they undergo slight subsidence. In this study, the subsidence of the primary key stratum and the sub key stratum were monitored to reach 1389 and 1437 mm, respectively. These values suggest that fractures were already formed in the main key stratum, and further, gave rise to ground subsidence under the influence of mining.

### Future research

This study focused on monitoring the movement and deformation of high-level and ultrathick key stratums in the overlying strata. The deep stratums were not taken as the primary monitoring objects. It is necessary to also monitor and analyze the movement of deep strata, such as the coal seam roof. Moreover, it is also important to monitor the movement and the stress in the coal pillar region outside the working face in the future research.

## Conclusions

The collaborative method of monitoring deformation before the working face reached the borehole (DOFS technology) and movement that working face surpassed the borehole (MPBX technology) was adopted, which has not been done in previous studies. We obtained the distance from the advance influence of mining on the ground surface as 219.2 m. Further, we observed the characteristics of the four-stage of high-level key stratum movement.

The upper region of the thick and hard stratum, as well as the interface between the thick stratum and the weak stratum, are prone to large deformations. The shallow strata are affected earlier than the deep strata. The deformation of the shallow strata occurred along the horizontal direction and the corresponding deformation magnitude is in general larger than that observed in the deep strata. This research result suggests that the angle of impact from advance mining is not a constant, and the boundary curve of the impact from advance mining is not a straight line with a fixed inclination.

The subsidence of the primary key stratum and the sub key stratum reached 1389 and 1437 mm, and their final relative displacement differed by 48 mm. This difference indicates an accumulative expansion effect that is induced by stress relief in the layered strata. No bed separation was found between the strata, and the key stratums tended to sink as a whole with the advancement of the working face.

This research provides an important reference to analyze the movement of thick high-level key stratums, and a reference for assessing the advanced influence range of mining in stratum and the protection of important infrastructure on the surface.
